# Application of decellularization methods for scaffold production: advantages, disadvantages, biosafety and modifications

**DOI:** 10.3389/fbioe.2025.1621641

**Published:** 2025-06-18

**Authors:** O. I. Shevchuk, V. V. Korcheva, N. S. Moskalenko, V. M. Kyryk, K. V. Kot, D. S. Krasnienkov

**Affiliations:** ^1^ D.F. Chebotarev Institute of Gerontology, National Academy of Medical Sciences of Ukraine, Kyiv, Ukraine; ^2^ Biology, V. N. Karazin Kharkiv National University, Kharkiv, Ukraine; ^3^ Institute of Genetic and Regenerative Medicine, National Scientific Center, M.D. Stazhesko Institute of Cardiology, Clinical and Regenerative Medicine, National Academy of Medical Sciences of Ukraine, Kyiv, Ukraine

**Keywords:** scaffold, decellularization, biocompatibility, scaffold modification, non-animal scaffold production

## Abstract

The development of efficient, biocompatible scaffolds is an actual challenge in tissue engineering. Scaffolds derived from animal sources offer promising structural and biochemical properties but require thorough decellularization to minimize immunogenicity and maintain extracellular matrix integrity. Effective decellularization requires a synergistic combination of methods to ensure complete removal of immunogenic cellular components while preserving critical extracellular matrix elements such as glycosaminoglycans, collagens, and growth factors. This review covers the application of some decellularization methods (physical, chemical) in scaffold production, highlighting their respective advantages, limitations, and biosafety considerations. Moreover, the importance of scaffold sterilization: both physical techniques like gamma irradiation and chemical agents–are mentioned for their efficacy and cytotoxic risks. Furthermore, scaffold modifications, particularly recellularization strategies, are discussed as key enhancements to improve biocompatibility and functional integration. Overall, the selection and optimization of decellularization protocols are crucial for the safe and effective clinical implementation of bioengineered scaffolds.

## Introduction

Decellularization is a technique that removes cells and immunogenic components from tissues and organs while maintaining the natural extracellular matrix (ECM) components. The decellularized matrix preserves the native microenvironment by maintaining the original tissue specific organization and structure, thereby providing an appropriate environment for cell function and differentiation (Amirazad et al., 2022; Vig and Fernandes, 2022). Decellularized extracellular matrix (dECM) serves as a natural scaffold in tissue engineering, as ECM is essential for tissue development (Gilbert et al., 2006). The extracellular matrix provides tissue mechanical structure through its network of molecules that support cellular repopulation and tissue remodeling. Several decellularization methods have been developed for reconstructing different tissue types and even entire organs (Engler et al., 2006).

The classification of decellularization techniques depends on the types of reagents used and the delivery methods involved (Table 1) (Uygun et al., 2010; Badylak et al., 2011; Crapo et al., 2011). Generally, tissue decellularization methods are divided into three main categories: chemical, biological, and physical methods. Perfusion-based systems or hybrid methods are applied to develop acellular scaffolds through these techniques (Crapo et al., 2011). dECM properties can be improved through the combination of decellularized matrix with additional molecules such as growth factors. dECM-based scaffolds that incorporate polycaprolactone (PCL) demonstrate enhanced capabilities for stem cell proliferation and migration. (Schenke-Layland et al., 2009; Bracaglia and Fisher, 2015).

**TABLE 1:** Classification of decellularization methods based on the types of reagents and delivery methods.

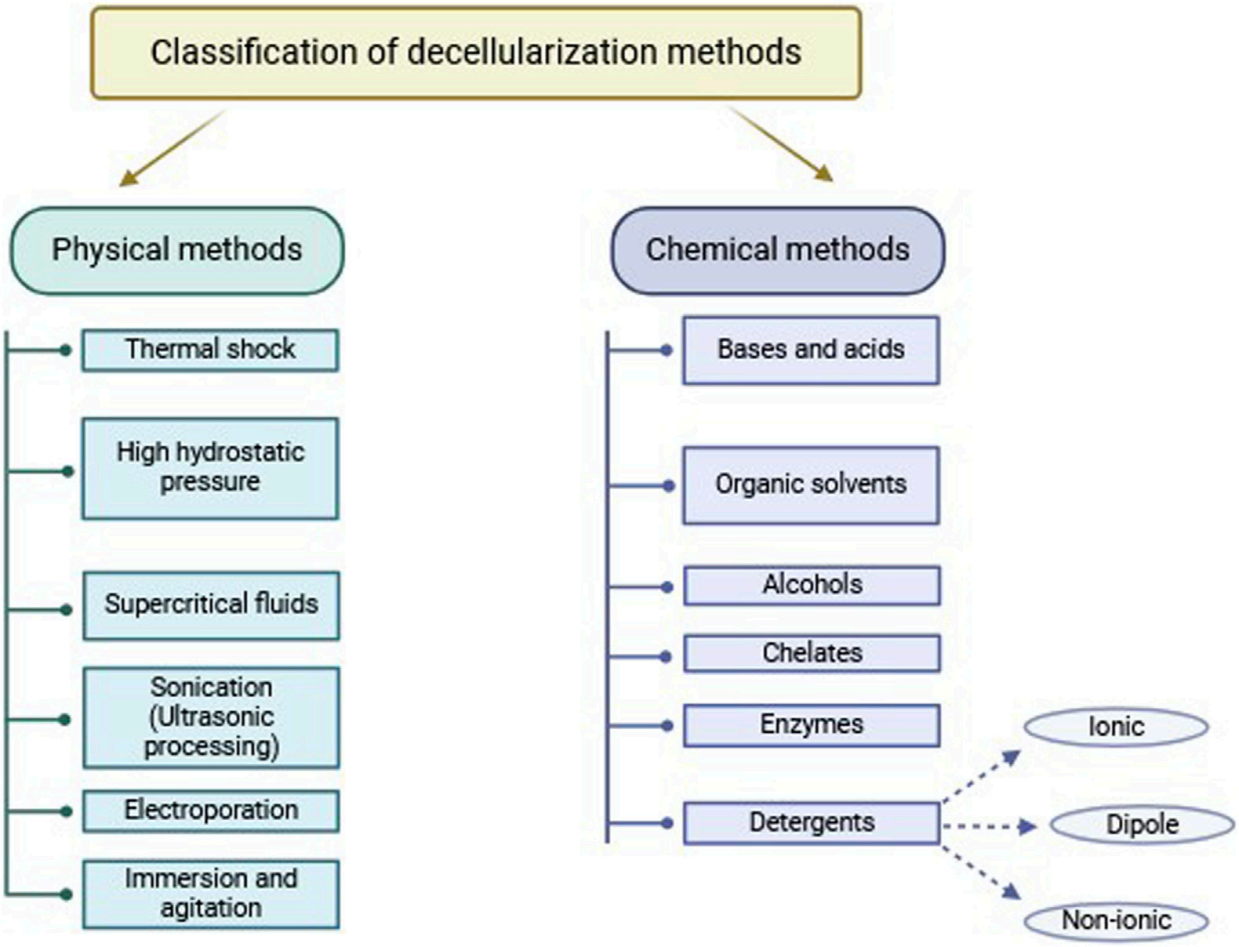

Another way of improving the properties of dECM-based transplants is genetic modification. Genetic modification may involve induction of apoptosis of stem cells during decellularization, which might be more effective without the use of harsh reagents that may disturb the mechanical and functional properties of the ECM. Additionally, genetic modification of stem cells used for recellularization can enhance transplant quality by enabling better control over their composition (Bourgine et al., 2014; Morris et al., 2018; Papadimitropoulos et al., 2015).

Decellularized materials are biocompatible and more stable than synthetic matrixes, making them widely used in various fields such as regenerative medicine and tissue engineering (Kawecki et al., 2018). Both acellular and recellularized decellularized tissues are predominantly applied as implantable scaffolds to promote the regeneration of lost or damaged tissues and organs (Mendibil et al., 2020). Given the prospect and growing use of dECM, a number of fabrication methods exist, including chemical solvent and alkali treatment, lyophilization, thermal shock, prototyping, stereolithography, modeling, selective laser ablation, 3D printing, bioprinting, cross-linking, and electrospinning (Eltom et al., 2019; Wasyłeczko et al., 2020).

This review will focus on possible decellularization methods, their advantages and disadvantages. Additionally, the article will consider some important parameters of decellularization process, such as temperature and sterilization methods. In order to cover all main aspects of d ECM manufacture, the review discuss some possible modifications of scaffolds, as well as the important issue of their biosafety.

## Physical methods of decellularization

Physical decellularization methods focus on removing cellular content by lysing cell membranes. The advantage of physical methods is that they offer even distribution of effect throughout the tissue alongside its more controlled and predictable action over protocols with chemicals or enzymes (Eltom et al., 2019). Another advantage of physical methods is its offering of an alternative to classical decellularization methods by greatly decreasing residual chemicals and enzymes (Pineda-Molina et al., 2024). However, it is important to note that physical methods alone are often insufficient for complete tissue decellularization. Therefore, they are frequently combined with chemical and enzymatic techniques to reduce exposure time and preserve the proteomic content of the ECM. Freeze-thaw cycles, high hydrostatic pressure (HHP), osmotic shock, ultrasonic treatment, and electroporation are among the widespread physical decellularization techniques (Table 2).

**TABLE 2:** Classification of physical methods of decellularization. Their main advantages and disadvantages.

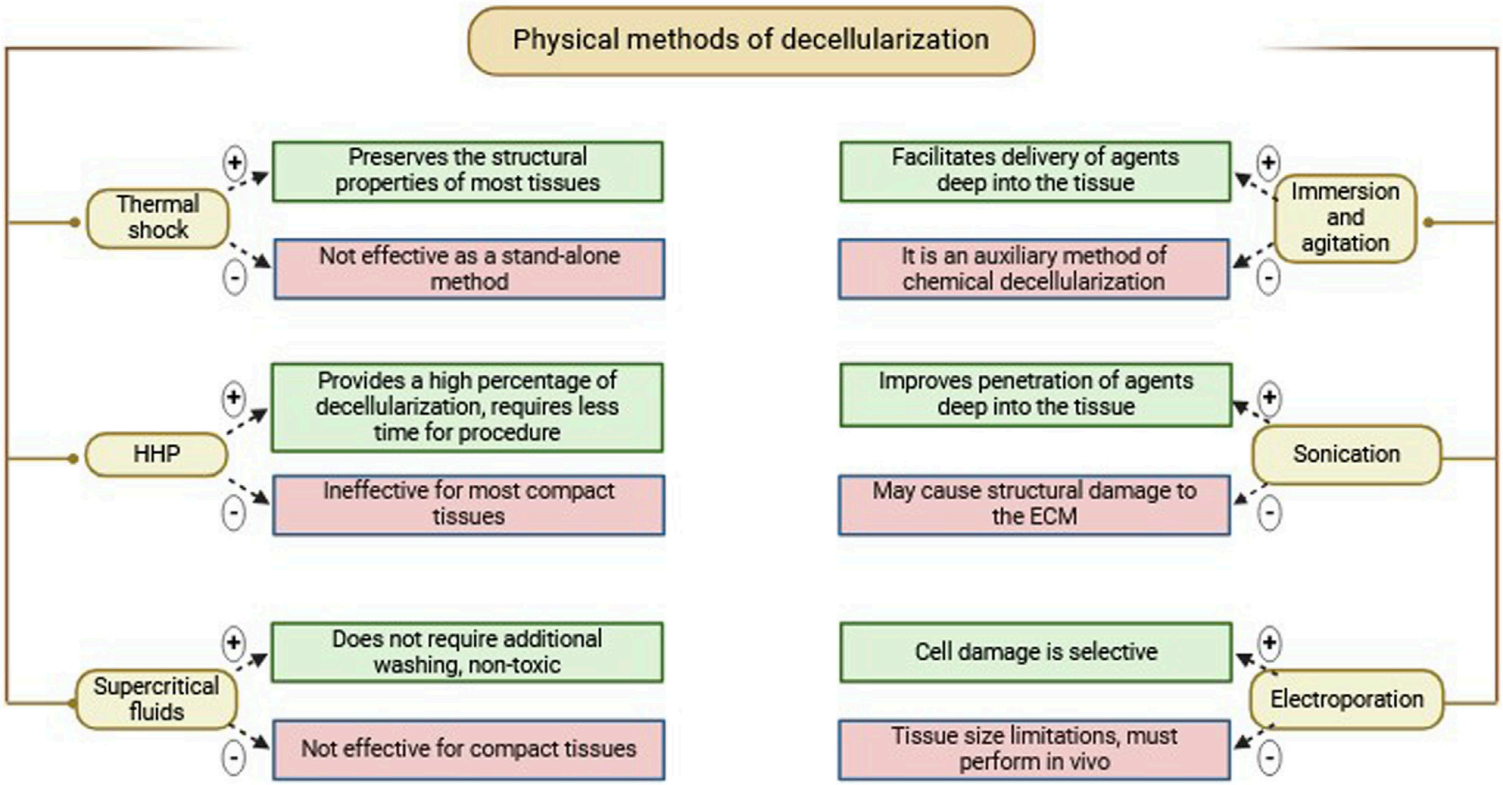

### Thermal shock

**FIGURE 1:**
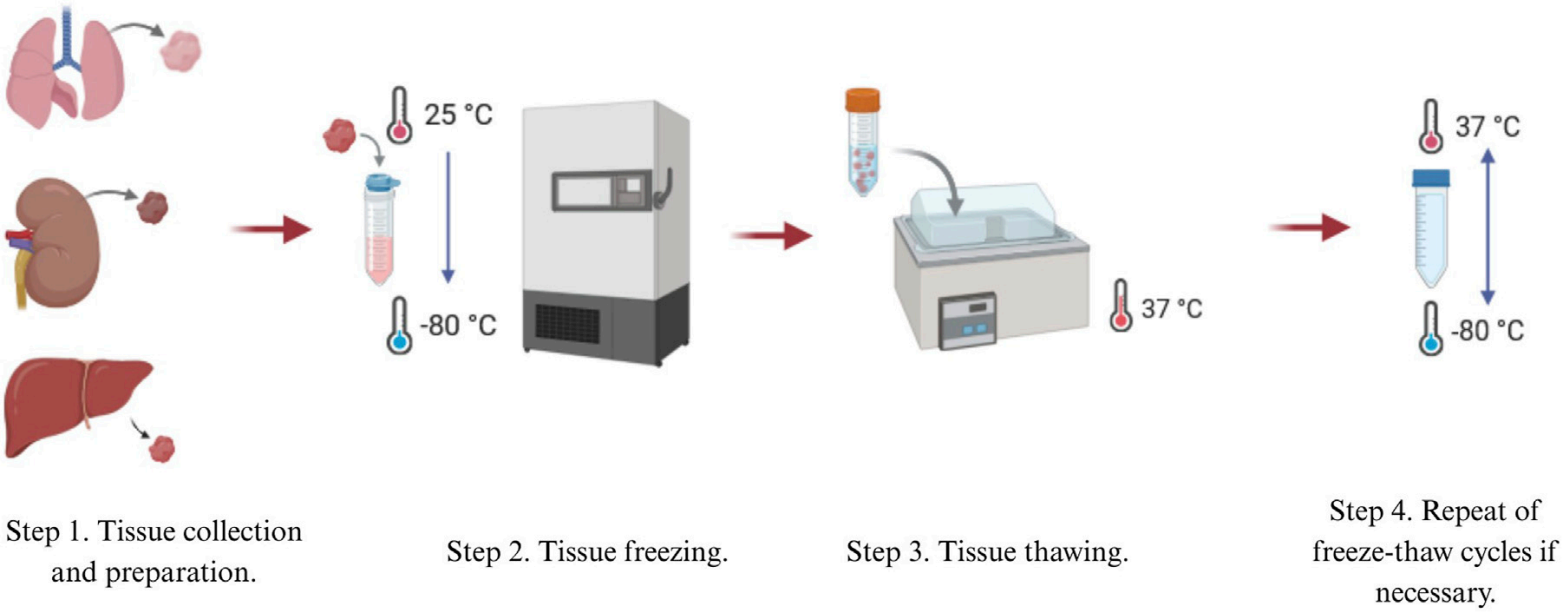
Scheme of tissues freeze-thaw cycling during decellularization process.

One of the most popular physical methods is thermal shock, or rapid freezing, usually utilized as the first step in the decellularization process. Thermal shock triggers cell lysis due to intracellular formation of ice crystals, which then destroys cell membranes (Mendibil et al., 2020). Freeze-thaw cycling of samples between ultra-low and biologically standard temperatures, generally between −80°C and 37°C, is employed (Figure 1). The number of freeze-thaw cycles can vary (Pineda-Molina et al., 2024; Mohsen et al., 2021). Thermal shock is also commonly used in decellularizing connective tissues such like ligaments, tendons, and nerve tissues (Gilbert et al., 2006). It has also been shown to decellularize cardiac scaffolds (Shahabipour et al., 2016) and liver tissues (Taylor and Sampaio, 2015).

Thermal shock efficiently kills cellular structures, but it is not an effective decellularization method by itself (Mohsen et al., 2021; Moffat et al., 2022). When fibroblast cell layers were subjected to three freeze-thaw cycles in experiments, collagen and elastin content, as well as mechanical properties, were maintained. However, 88% of DNA content remained in the cells layers (Gilpin and Yang, 2017), implying that immune rejection could result from using this process alone. Thus, further treatments are needed to eliminate remaining cellular material (Mohsen et al., 2021; Moffat et al., 2022).

Although not extensive, thermal shock enhances decellularization efficiency when combined with other methods. In a study on the decellularization of large tendons, thermal shock was used in combination with Triton X-100 and sodium dodecyl sulfate (SDS). Freeze-thaw cycles improved the removal of DNA and nucleic acids by 20% compared to chemical decellularization alone (Mohsen et al., 2021; Burk et al., 2014a).

Freeze-thaw cycles do not significantly alter ECM mechanical properties and produce only minimal disruptions on tissue ultrastructure. The utilization of cryoprotectants also minimizes harmful effects (Keane et al., 2015). However, control of ice crystal size through temperature regulation is necessary to prevent excessive ECM damage (Mendibil et al., 2020).

Thermal shock may be applied to numerous tissue types. Despite being commonly used for nerve tissue decellularization, it has been reported that freeze-thaw cycles may damage nerve ultrastructure, though mechanical properties remain intact (Hsu et al., 2023). Thermal shock also preserves the elastic modulus of decellularized kidneys, with no porosity increase or structural damage detectable by scanning electron microscopy (SEM) (Mohsen et al., 2021; Poornejad et al., 2015). Thermal shock thus maintains structural integrity in most tissues and is a widely used decellularization method. Its relatively gentle nature, however, may be insufficient for foreign DNA removal.

### High hydrostatic pressure

HHP is a physical decellularization method that disrupts cell membranes and alters ultrastructure, enhancing cell lysis and the efficacy of chemical or enzymatic treatment. The method increases intercellular pressure, which facilitates easier removal of cellular residues (Mendibil et al., 2020).

This method involves the application of pressurized water to tissue, with this speeding up decellularization time and reducing the time of exposure relative to enzyme- or detergent-based methods. Ice crystal damage to ECM structures is however induced by water presence. Temperatures are elevated in HHP decellularization in order to prevent ice crystal formation but induce higher entropy that can compromise ECM integrity. Colloidal agents such as dextran are used to minimize such effects (Crapo et al., 2011; Mohsen et al., 2021).

HHP was also applied successfully in some decellularization processes. Porcine retina was completely decellularized with 980 MPa for 10 min, for instance (Hashimoto et al., 2010). The same approach was used for porcine aorta decellularization, where 4-week post-transplant observations showed that the decellularized vessel kept up blood pressure and prevented thrombosis (Funamoto et al., 2010). These studies demonstrated HHP’s effectiveness in releasing cellular components, although it requires combination treatments to achieve complete decellularization. With the combination of chemical washing, full decellularization was accomplished and was as effective as detergent-based methods (Hashimoto et al., 2010).

Such technique, which is optimal in less compactly structured tissues of lung and liver (Gilbert et al., 2006), has fewer applications practically despite advantages of reduced time of decellularization in addition to retention of structure in the ECM and its immunocompatibility boosted by the immune system.

### Supercritical fluids

Supercritical fluids are liquids that exist at pressures and temperatures above their critical points with low viscosity and high diffusivity. Carbon dioxide (CO_2_) can be rapidly removed from tissues without requiring additional rinsing procedures. Decellularization is performed by controlled flow of the fluid through tissues, as in critical point drying, enabling complete removal of cellular residues (Pineda-Molina et al., 2024). Moreover, CO_2_ has been shown to decellularize tissues within a timeframe comparable to that required for most chemical agents. Carbon dioxide, the gas employed in this technique, is an appropriate gas for decellularization due to its non-toxic, non-flammable, and relatively inert nature, along with being inexpensive (Moffat et al., 2022; Hsu et al., 2023). One of the key advantages of supercritical fluid decellularization is the minimal alteration of the mechanical properties of ECM and the exclusion of the lyophilization step as a preparatory step for ECM storage and processing (Gilbert et al., 2006).

### Immersion and agitation

One of the most commonly used methods for tissue decellularization involves incubation in chemical, detergent, and/or enzymatic solutions with mechanical agitation (Keane et al., 2015). The tissue samples are immersed in a decellularizing solution, allowing free movement of the tissue and full contact with the chemical agent, which allows for uniform treatment of the entire surface and depth of the sample (Pineda-Molina et al., 2024). Protocols based on this decellularization method have been applied to numerous tissues like the bladder, esophagus, trachea, skeletal muscle, heart valves, peripheral nerves, spinal cord, cartilage, and dermis. The duration of each protocol is determined by factors like the strength of agitation, degree of mechanical trauma, concentration and type of chemical reagent, detergent, or enzyme, as well as tissue thickness and density. For instance, thin tissues like the bladder or small intestine can be decellularized within a short period of time (one to two h) and the efficiency of cell removal varying with the agitation intensity. Dense tissues like the dermis and trachea require longer treatment (12–72 h) and the application of enzyme, alcohol, or detergent combinations with continuous agitation (Keane et al., 2015). Mechanical agitation can be achieved with the assistance of a magnetic stirrer, orbital shaker, or low-profile roller (Gilbert et al., 2006).

### Ultrasound treatment (sonication)

Ultrasound treatment, or sonication, enhances the penetration of decellularizing agents into the tissues. Ultrasound treatment has been successfully utilized to decellularize tissues such as the aorta, arteries, larynx, and cartilage. The intensity of cavitation that occurs during ultrasound treatment depends upon parameters such as pH, temperature, viscosity, diffusion rate of dissolved oxygen, vapor pressure, and gas solubility within the liquid medium. These conditions vary greatly based on the concentration of the decellularizing agent. Ultrasound treatments generally employ low concentrations of SDS since this detergent effectively solubilizes cellular and matrix components (Moffat et al., 2022). However, ultrasound treatment is a physically stressful process that may cause structural damage to the ECM, although such risk depends on the specific conditions applied. Ultrasound baths provide more uniform penetration of chemical agents into the tissue and are less disturbing to ECM ultrastructure compared to ultrasonic homogenizers, but require a longer duration of processing (Moffat et al., 2022). Sonication thus is not an isolated decellularization procedure but is used primarily as an auxiliary step. Ultrasound treatment, often combined with chemical agents such as SDS, shows high efficiency in removing cellular components and DNA, achieving over 90% of cell destruction (Lin et al., 2021).

Ultrasound preserves the architecture of collagen fibrils, although sometimes there is a loss of sulfated glycosaminoglycans (GAGs) and the formation of micropores, which can negatively affect the viscoelastic properties of the matrix (Azhim et al., 2014).

### Electroporation

**FIGURE 2:**
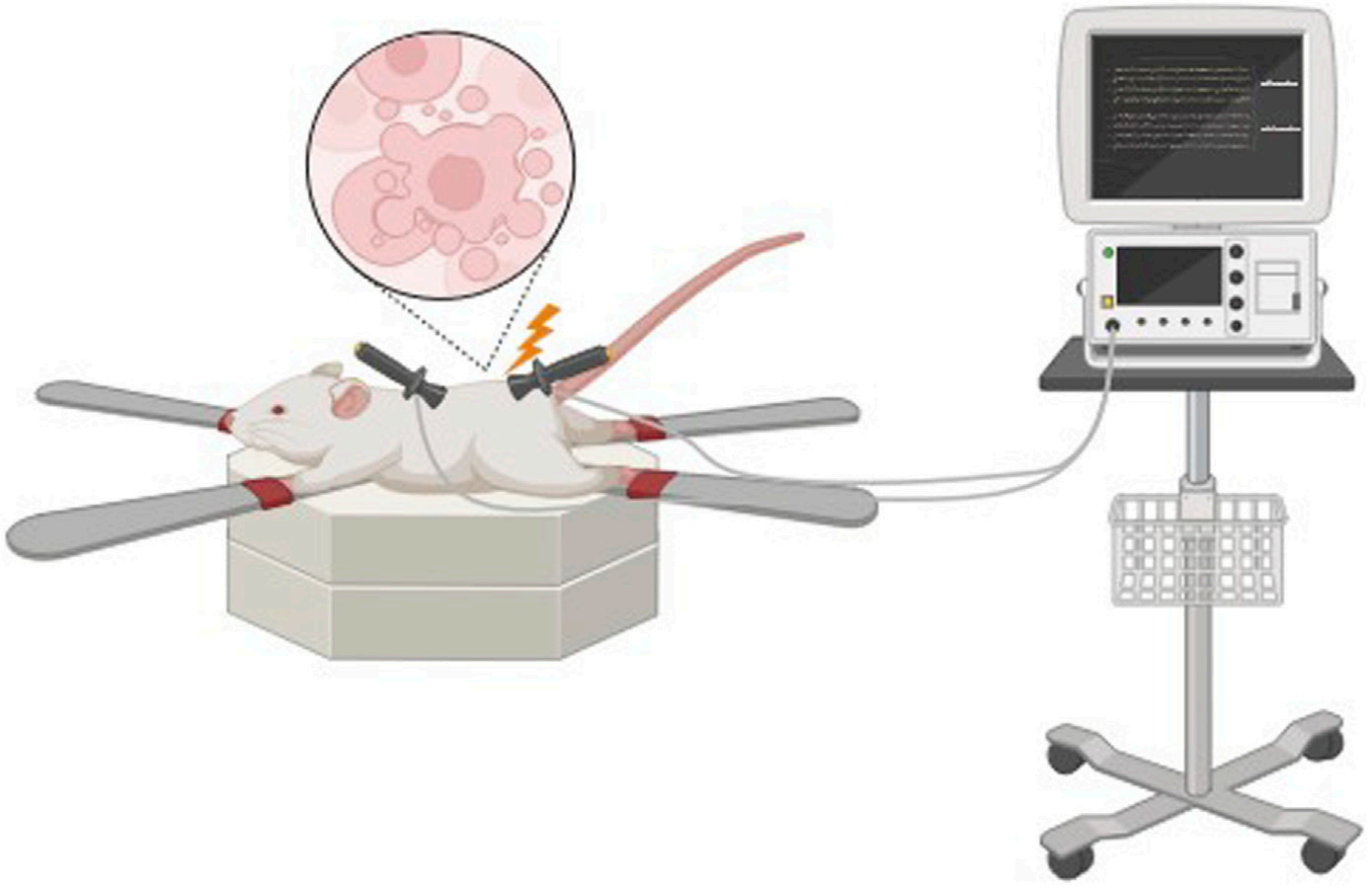
Cell lysis during *in vivo* electroporation.

Non-thermal irreversible electroporation (NTIRE) is based on the application of microsecond electrical pulses through the tissue to form micropores in the cell membranes, resulting in cell lysis (Figure 2).

Micropores result in cellular homeostasis disruptions, resulting in cell death, which can be used effectively in a range of decellularization protocols (Pineda-Molina et al., 2024). Cell damage in irreversible electroporation is selective, and temperature control during the process preserves minimally denatured ECM and tissue architecture. The advantage of NTIRE is that it does not cause thermal damage to the entire tissue and preserves the ECM structure. Also, since no chemical reagents are used, it reduces the risk of residual toxicity and simplifies the cleaning process (Sano et al., 2010). In addition, in order to prevent heat damage, it creates a distinct area of tissue ablation with distinct, cell-scale boundaries separating the impacted and unaffected areas (Phillips et al., 2010). Therefore, electroporation has prospects for decellularization of whole organs.

A drawback of this method is limited electrode size, which significantly limits the permissible tissue size for decellularization and this is the main limitation for NTIRE decellularization of large organs. Additionally, decellularization has to be performed *in vivo* to avoid inflammatory responses because of the immune system (Mohsen et al., 2021). This method was first used for *in vivo* decellularization of rat carotid arteries. The results demonstrated that cellular components were progressively removed from the tissue over 3 days (Phillips et al., 2010). In 2018, Zhang et al. (Zhang et al., 2018) used NTIRE to treat rat liver tissue and investigate the histological and molecular events occurring within 24 h post-treatment. The main findings were that, 6 hours after NTIRE, complete hepatocyte breakdown within an intact extracellular matrix was observed, and 24 h later, new hepatocytes appeared at the treatment site. These results suggest that 24 h after NTIRE is an optimal time point for implantation. The researchers also found that, contrary to common claims in the NTIRE literature, there is no evidence that NTIRE induces apoptotic cell death.

The degree of decellularization during the electroporation process may be tuned through the modification of pulse duration, frequency, the number of pulses, and the electric field intensity (Mohsen et al., 2021).

To sum up, the most universal physical method being used within protocols of decellularization is thermal shock. However, none of the presented methods can be used independently, as they primarily serve as auxiliary steps in cellular content removal from tissues.

### Chemical methods

Similar to physical methods, chemical decellularization methods are also designed to induce cell lysis. In this case, the lytic agents are various chemical compounds, such as detergents, acids, bases, or enzymes (Table 3).

**TABLE 3:** Classification of chemical agents used for decellularization.

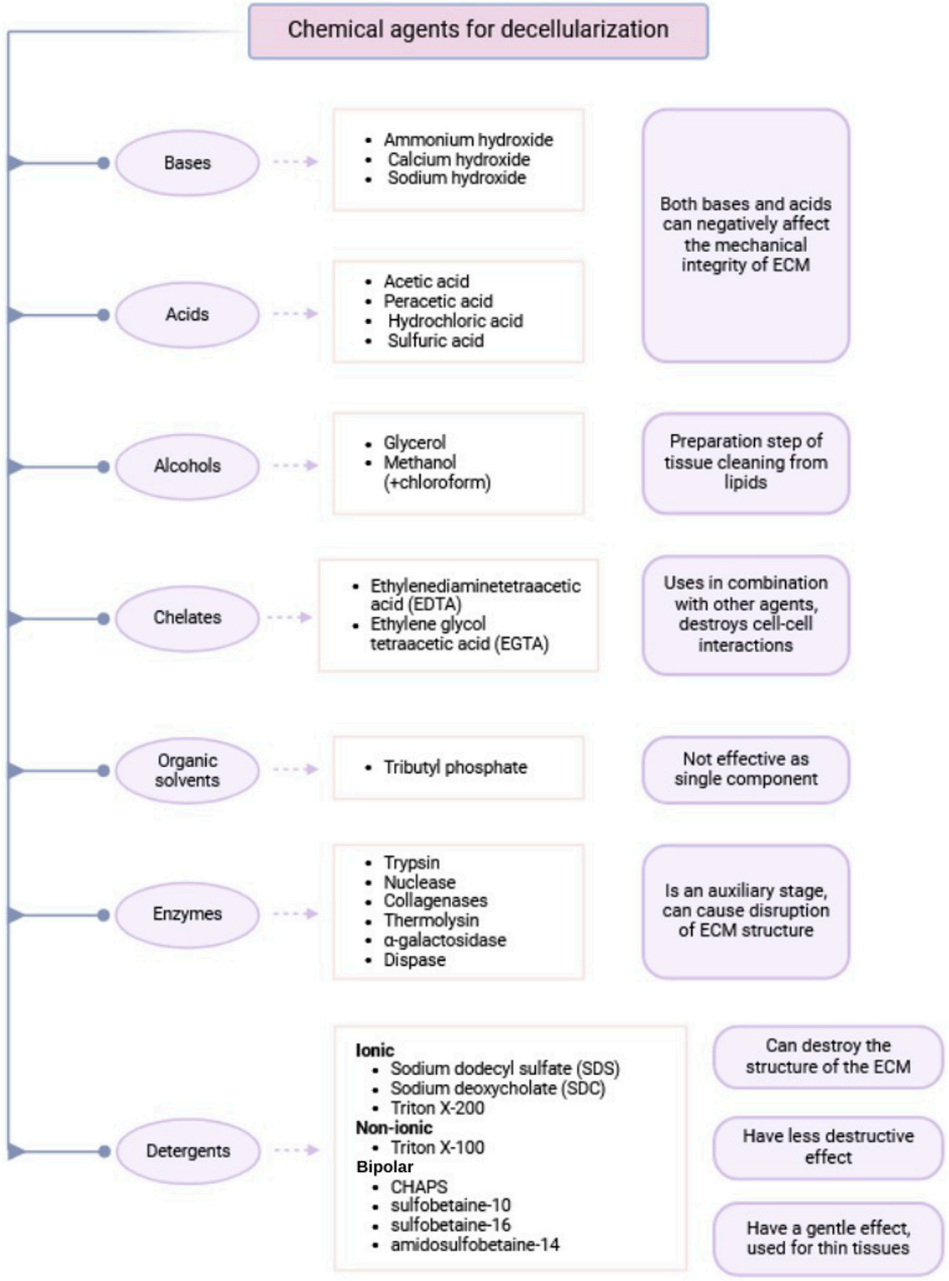

Chemical compounds used in decellularization protocols must meet specific criteria. When choosing these compounds, it is required to ensure that the chemical decellularization process does not degrade ECM components while being effective at eliminating the cellular component and nuclear material. Also, the choice of protocol must take into consideration the specific characteristics of the tissue or organ because the composition of the ECM varies according to its origin. The key steps in the decellularization process are the disruption of cell membranes, solubilization of cellular components, and removal of nucleic acids. (Damodaran and Vermette)

Chemical agents used for ECM extraction can be classified into the following classes: acids and bases, alcohols, cholates, organic solvents and detergents. Detergents are further classified into ionic, nonionic, and amphoteric types. Each of these classes possesses distinct advantages and disadvantages, which are explained below.

### Acids and bases

The primary mechanism of action of acids and bases in decellularization is hydrolysis of cytoplasmic components, degradation of nucleic acids, and protein denaturation (McInnes et al., 2022; Zahmati et al., 2017). However, both types of compounds can adversely affect the mechanical stability of the ECM (Damodaran and Vermette). Therefore, the selection of appropriate reagents and optimizing the exposure time are the primary determinants of preserving ECM properties.

Among the most commonly used acids in decellularization protocols are acetic acid, peracetic acid (PAA), hydrochloric acid, and sulfuric acid. Acetic acid was suggested to reduce the tensile strength of the ECM without inhibiting the proliferation of human mesenchymal multipotent stem cells, showing a higher level of biocompatibility (Dong et al., 2009). On the other hand, PAA has lower impact on ECM mechanical properties but fails to decellularize efficiently (Damodaran and Vermette). PAA retains the vital ECM components such as transforming growth factor-β (TGF-β), laminin, and fibronectin, which play a crucial role in cell adhesion (Zahmati et al., 2017). Due to its low efficacy in decellularization, PAA is largely used as a disinfectant, often mixed with ethanol. As a strong oxidizing agent, it is bactericidal, viricidal, fungicidal, and sporicidal properties in nature and can be used for scaffold sterilization (McInnes et al., 2022).

Typical bases used in decellularization protocols are ammonium hydroxide, calcium hydroxide, and sodium hydroxide (Zahmati et al., 2017). They are used during decellularization uniquely as they break down the collagen matrix and growth factors (Zahmati et al., 2017), with negative impacts on downstream recellularization and biocompatibility as a whole. Furthermore, bases such as sodium hydroxide can be cytotoxic if not thoroughly washed out (Damodaran and Vermette). In practical applications, bases are primarily used to remove traces of hair from tissue samples before decellularization.

Overall, acids and bases are hard to remove completely from scaffolds, and their residual traces can have adverse effects not just on ECM components but also on the cells used during recellularization step (McInnes et al., 2022). Thus, their use will reduce the efficiency of recellularization and result in possible biocompatibility problems.

### Alcohols

Alcohols are typically used in the decellularization preparatory phase to facilitate easier lipid extraction from tissues. Lipid-poor tissues are better decellularized because cell lysis and subsequent washing off by the ECM are easier (Albanna and Holmes, 2016).

Glycerol is one of the most commonly used alcohols for delipidating of biological scaffolds. Glycerol selectively removes lipids, possesses lytic activity, and causes dehydration of the tissue (Zahmati et al., 2017; Flynn, 2010). Methanol is another common alcohol to delipidate tissues and is typically applied with chloroform (Flynn, 2010).

The primary limitation of alcohols in scaffold preparation protocols is that they precipitate proteins (Zahmati et al., 2017). This can disrupt the structural integrity of ECM proteins, with consequent loss of mechanical integrity in the scaffold. However, alcohols can also be considered as anti-calcification agents since they are utilized to extract lipids responsible for tissue calcification (Zahmati et al., 2017).

### Chelating agents

Chelating agents, or chelators, are chemical compounds formed by the binding of a ligand to an ion of metal (Jung et al., 2022). In biological applications, they act by targeting the toxic metal component, inhibiting enzymes such as metalloproteinases, and induce disruption of cell adhesion (McInnes et al., 2022). Classic examples of chelating agents used in decellularization include ethylenediaminetetraacetic acid (EDTA) and ethylene glycol tetraacetic acid (EGTA).

Chelators can break intercellular connections by sequestering essential adhesion ions such as Ca^2+^ and Mg^2+^ (Zahmati et al., 2017). However, they are never used alone in decellularization protocols but alongside in combination with other compounds, such as trypsin (Zahmati et al., 2017; Blaudez et al., 2019), because chelators alone cannot completely dissociate ECM. Additionally, improper exposure times during EDTA treatment can disrupt the mechanical framework of the ECM (Atala, 2005).

### Organic solvents

The use of organic solvents in decellularization protocols is not common. Agents of this kind can be utilized as a substitute for ionic and nonionic detergents. Additionally, some organic solvents possess antiviral properties (Zahmati et al., 2017).

Tributyl phosphate (TBP) is a widely used organic solvent in decellularization, functioning as a disruptor of tissue protein components (Zahmati et al., 2017). The main advantage of TBP is its relatively mild effect, which helps preserve the mechanical integrity of the ECM. However, this gentle nature can also limit its effectiveness in the complete removal of cellular components (Zahmati et al., 2017; Blaudez et al., 2019).

### Enzymes

Enzymes, being the biological catalysts composed primarily of proteins, are also utilized in decellularization protocols. The most commonly used enzymes are trypsin, nucleases, collagenases, thermolysin, α-galactosidases, and dispase (Damodaran and Vermette).

The action of DNases and RNases hydrolyze genetic material in such a manner to break down DNA but not damage the structure of ECM (McInnes et al., 2022). The enzymes are found to be difficult to eliminate completely from tissue and only function well with agents that destabilize membrane structures (Blaudez et al., 2019). A significant disadvantage with nucleases is their poor diffusion into dense tissues such as tendons and bones which could lead to heterogeneous decellularization. In such cases, DNA content may be elevated in the deeper layers of tissues with tolerable content on the periphery (Burk et al., 2014b). Notably, the permissible residual DNA content in decellularized ECM must not exceed 50 ng/mg (McInnes et al., 2022). Therefore, nucleases are typically applied after stronger agents, such as detergents, to ensure thorough removal of foreign nucleic acids.

The most common enzyme that is used in decellularization protocols is trypsin, which is an extremely selective proteolytic enzyme that degrades peptide bonds, disrupting cell adhesion and degrading tissue structure (Crapo et al., 2011; McInnes et al., 2022). Trypsin is typically used in combination with chelating agents, predominantly EDTA, which further affects intercellular relationships (Blaudez et al., 2019). Complete decellularization cannot be achieved through the application of enzymes alone (Crapo et al., 2011). For example, after incubation of organs or tissues for 24 h at 37°C in a 0.05% trypsin-EDTA solution (Gungormus et al., 2015), secondary incubation in acids and detergents, such as Triton X-100 (24 h at room temperature) (Morris et al., 2018), or in a combination of acids and detergents (e.g., 1.5% peracetic acid +2% Triton X-100 for 4 h at 37°C) (Whitlock et al., 2007), is usually required.

Trypsin was studied (Ghassemi et al., 2019) as one of the key components of the cartilage decellularization protocol. In the first phase of the experiment, it was shown that trypsin is able to effectively destroy cell nuclei - almost complete degradation was observed at a concentration of ≥0.25%. At the same time, the tissue lost structural density and acquired a gel-like consistency, which indicates potential damage to the ECM with excessive enzyme action. The combination of trypsin with EDTA (a chelating agent) contributed to better washing out of cell debris than trypsin alone. In the final optimized protocol, the use of 0.05% trypsin/EDTA for 1 day, followed by treatment with 3% SDS (day 2–3) and 3% Triton X-100 (day 4–5), ensured almost complete decellularization.

Protocols involving trypsin achieved efficient decellularization in a significantly shorter time compared to standard methods, while maintaining high cell viability and scaffold functionality after reseeding with chondrocytes (Giraldo-Gomez et al., 2016). These results are consistent with previous reports indicating that partial loss of ECM does not prevent the successful application of trypsin-treated scaffolds.

Thus, enzymatic decellularization is itself an ineffective methodology. Instead, enzymes are incorporated as an auxiliary step, facilitating the breakdown of cellular junctions to enhance detergent penetration into the tissue. Additionally, not enough or very aggressive enzymatic incubation is capable of causing compromise to the mechanical integrity of the ECM.

### Detergents

**FIGURE 3:**
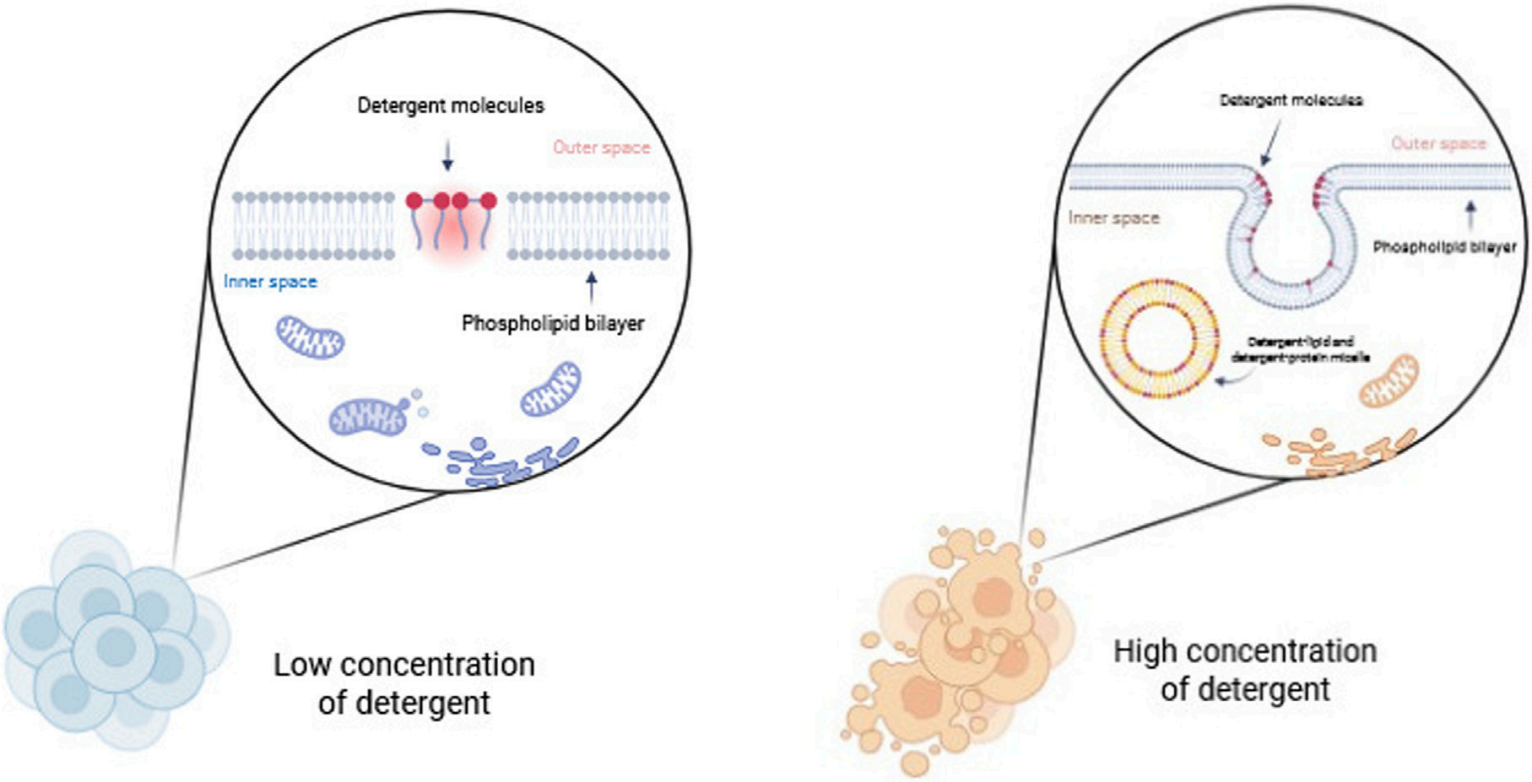
Disrupting cellular membrane by detergents.

Detergents are amphipathic molecules composed of both polar and hydrophobic groups. Detergents are structurally defined by a polar head and a hydrophobic long carbon chain, or nonpolar tail. This class of compounds disrupts cellular membranes by solubilizing them, forming an imitation of the lipid bilayer environment. The dissolution of biological membranes by detergents occurs in several stages. At low concentrations, detergents bind to the membrane, disrupting the lipid bilayer. At higher detergent concentrations, the membrane becomes saturated and is broken up into mixed micelles of membrane lipids and detergent. Eventually, detergent-lipid mixed micelles and detergent-protein micelles are formed, and the membrane is essentially dissolved (Figure 3) (Bhairi and Mohan, 2007).

Detergents are classified based on the nature of their polar head groups into three main classes: ionic, nonionic, and zwitterionic (dipolar ionic) detergents (Zahmati et al., 2017).

Ionic detergents include reagents such as SDS, sodium deoxycholate (SDC), sodium lauryl sulfate, sodium lauroyl sarcosinate, potassium laurate, and Triton X-200 (McInnes et al., 2022). Ionic detergents are widely used in decellularization processes for solubilizing and removing membrane proteins and protein-associated DNA (Zahmati et al., 2017).

SDS is one of the most commonly used detergents, which has been found to be effective in removing cellular debris and nuclear and cytoplasmic components from compact and dense tissues such as the heart and bones (Wang et al., 2012). Despite being highly effective, its utilization is unfavorable to the structure of the ECM (Zahmati et al., 2017). Reduction of glycosaminoglycans (Blaudez et al., 2019) and ECM growth factors (Zahmati et al., 2017) has been observed following SDS treatment. Due to its high affinity for proteins, SDS disrupts protein-protein interactions, leading to degradation of the collagen matrix and mechanical disruption of the integrity of the scaffold. The extent of ECM damage is directly related to the concentration and incubation period of SDS (Zahmati et al., 2017). The washing process is another important consideration for SDS use in decellularization, as SDS residues can lead to inflammation and fibrosis both *in vitro* and *in vivo* (Friedrich et al., 2018). In addition, residual SDS disrupts cell viability during recellularization due to its strong cytotoxicity (Ghorbani et al., 2022). To improve scaffold quality, SDS can be precipitated out with CaCl_2_ (Friedrich et al., 2018), and incubation time and detergent concentration can be optimized to preserve ECM structural and functional molecules (McInnes et al., 2022). These findings indicate that while SDS is highly effective, it is far from an ideal decellularization agent.

SDC is another ionic detergent used for tissue and organ decellularization. It has more potent and disruptive effect on ECM than SDS (Zahmati et al., 2017). Upon contact with the cell membrane, SDC inserts its cholate moiety into the lipid bilayer, creating pores that ultimately lead to membrane disruption (Damodaran and Vermette). SDC is typically combined with other amphiphilic detergents (Zahmati et al., 2017). Despite its aggressive action, SDC, by itself or combined with other detergents, has proven to be efficient in preserving key ECM properties after decellularization (Rieder et al., 2004; Sabetkish et al., 2015).

Triton X-200 is another ionic detergent that can be used for decellularization but is less effective and has similar effects to SDS and SDC (Zahmati et al., 2017). The extent of ECM damage caused by detergents relies on detergent type, exposure time, tissue type, and donor age (Zahmati et al., 2017). Ionic detergents are part of the majority of protocols in combination with other chemical agents. Therefore, when selecting ionic detergents for decellularization, their advantages should be considered along with their potential negative impacts on ECM.

At the same time, non-ionic detergents show a less destructive effect on the ECM (Zahmati et al., 2017). Their mechanism of action is based on the disruption of lipid-lipid interactions, but while ionic detergents interfere with protein-protein interactions, non-ionic detergents do not (Seddon et al., 2004).

Triton X-100 is commonly used as non-ionic detergent in decellularization protocols and its application is as common as the use of SDS (McInnes et al., 2022). By binding to polar head groups, Triton X-100 destabilizes lipid bilayer hydrogen bonds, leading to disruption of the cell membrane (Koley and Bard, 2010). However, Triton X-100 has a relatively poor ability to remove cells from most tissue types, e.g., veins, corneas, urethras, hearts, and kidneys (Damodaran and Vermette). As a result, it is largely applied in combination with other agents, such as SDS, or as a blend with supplementary methods for maximizing cell removal and efficient ECM decellularization. Despite its milder action, Triton X-100 has severe side effects that could impact ECM integrity. Triton X-100 has been found in certain research to be able to remove glycosaminoglycans from the ECM surface in certain tissues (Bader et al., 2000). Therefore, careful optimization of Triton X-100 concentration and incubation time is necessary, as its relatively weak action can result in incomplete decellularization.

Zwitterionic or bipolar detergents exhibit characteristics of both ionic and non-ionic detergents. These compounds have a net charge of zero and possess hydrophilic and hydrophobic regions. Members of this category include 3-([3-cholamidopropyl]dimethylammonio)-1-propane sulfonate hydrate (CHAPS), sulfobetaine-10, sulfobetaine-16, and amidosulfobetaine-14 (McInnes et al., 2022). Their primary mechanism of action is to interfere with protein-protein interactions, as ionic detergents, but less severely. Since they are comparatively mild in action, bipolar detergents are employed primarily for the decellularization of thin tissues (Zahmati et al., 2017).

CHAPS is widespread detergent used in decellularization. Unlike most ionic detergents, CHAPS does not create pores in the cell membrane but can still disrupt the lipid bilayer structure (Damodaran and Vermette). It is more acceptable for preserving the mechanical strength of the ECM and has been used successfully in lung (O’Neill et al., 2013) and blood vessel decellularization (Dahl et al., 2003). Although CHAPS effectively removes whole cells, some studies indicate the potential for preserving cytoplasmic proteins and DNA fragments (Tsuchiya et al., 2014). Compared to other detergents, such as sodium dodecyl sulfate (SDS), CHAPS may leave higher levels of residual DNA, which is a critical factor for scaffold immunogenicity (Tsuchiya et al., 2014). This means that additional washing steps or the combination of CHAPS with other methods may be required to ensure total biocompatibility.

The pH level of the CHAPS solution has a significant effect on the efficiency of DNA removal. Studies have shown that DNA content is significantly higher in tissues decellularized at pH eight and pH 10 compared to pH 12 (Fernández-Pérez and Ahearne, 2019). This suggests that higher pH promotes better DNA removal, likely due to increased protein denaturation and nucleic acid degradation in an alkaline environment. Therefore, optimization of pH is critical to achieve the desired level of decellularization. A key advantage of CHAPS is its non-denaturing properties, which allow the structural integrity and biochemical composition of the ECM to be preserved (Rieder et al., 2004; Tsuchiya et al., 2014). Studies have confirmed that CHAPS does not damage collagen and elastin structures, which is important for maintaining the compliance and elasticity of tissues, particularly the lungs (Rieder et al., 2004; Tsuchiya et al., 2014).

Other compounds such as sulfobetaine-10 and sulfobetaine-16 have also been applied in nerve tissue decellularization (Hudson et al., 2004).

### Decellularization temperature conditions

Temperature may also be a key factor in determining decellularization success, since temperature fluctuations can affect the structure and composition of ECM proteins. Temperature is, nonetheless, most crucial in physical decellularization methods, including freeze-thawing and HHP. For instance, in corneal decellularization by HHP, temperatures of 10°C better maintained collagen and glycosaminoglycans compared to higher temperatures of 30°C (Gilpin and Yang, 2017). A different study (Negishi et al., 2011) investigated HHP decellularization of the carotid artery and contrasted washing temperatures for the produced ECM. The results showed that 37°C washing degraded collagen, but washing at 4°C maintained collagen content and conformation. In syngeneic rat carotid artery transplantation, 37°C washed arteries occluded after 2 weeks, while 4°C washed arteries were patent. This indicates that collagen denaturation within decellularized arteries affects *in vivo* function.

Tissue preservation is another temperature-dependent factor in ECM scaffold production. Freezing tissues leads to intracellular ice crystal formation, causing membrane damage and facilitating chemical reagent penetration, thus enhancing cell removal regardless of the initial decellularization solution used (Miranda et al., 2021). Freeze-thaw cycles are not harmful to ECM ultrastructure. Lower storage temperatures allow more extended preservation; long-term preservation is optimal at −80°C while short-term preservation is sufficient at −20°C.

The effectiveness of physical decellularization methods involving freeze-thaw cycles depends on temperature, which has been discussed in detail in the respective section. Chemical decellularization methods typically utilize standard room temperature (20°C–25°C) (Miranda et al., 2021). Temperature variations are acceptable with enzymatic treatment, e.g., trypsinization. Trypsin can be used in a cold protocol (4°C) or incubated at 37°C. Cold trypsinization requires longer exposure periods and higher enzyme concentration, while conventional trypsinization requires shorter exposure periods and lower enzyme concentration to prevent excessive degradation of the ECM (Gungormus et al., 2015; Whitlock et al., 2007).

### Approaches of scaffold sterilization

Sterilization methods are commonly divided into two groups: physical and chemical sterilization methods (Table 4).

**TABLE 4:** Classification of sterilization methods used in biomedical system.

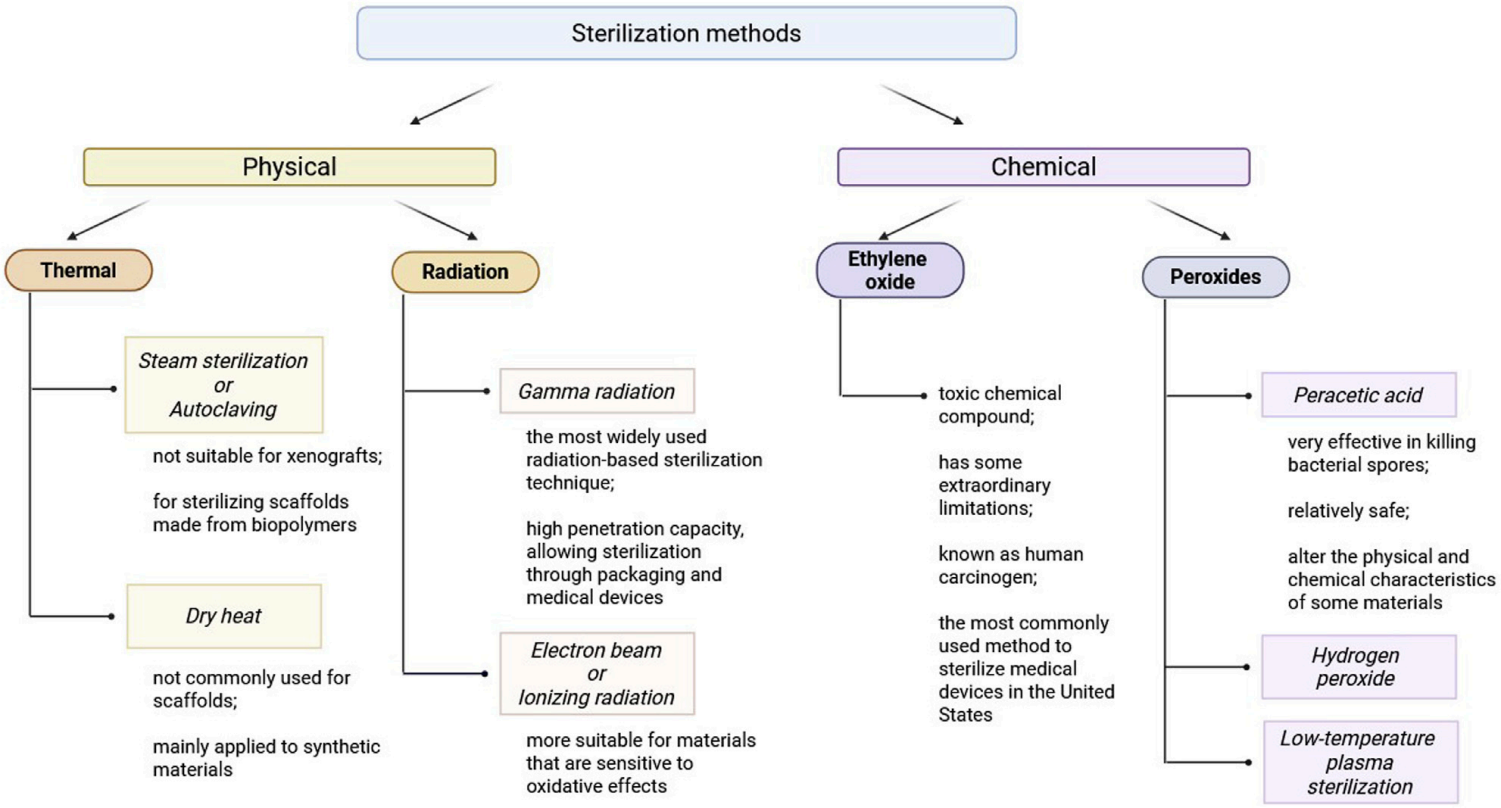

### Physical sterilization methods

The application of high temperatures is the most commonly used sterilization method, particularly in healthcare settings. Alternative methods should only be considered if the material is unable to withstand elevated temperatures (Fracalossi Rediguieri et al., 2016).

Steam sterilization or autoclaving is highly effective in decontaminating complex surfaces with deep recesses and sharp edges due to direct exposure to pressurized saturated steam. The combination of heat and moisture makes autoclaving a widely used sterilization method. A standard sterilization cycle operates at 121°C for 15–20 min or at 132°C for 4 min in a pre-vacuum sterilizer (World Health Organization, 2011). The thickness and porosity of scaffolds must be carefully considered when choosing autoclaving or other direct-contact sterilization methods. Porous materials require special processing parameters, such as pre-vacuum cycles, to remove trapped air and ensure complete steam penetration. Tissue engineering scaffolds generally feature high porosity, which should not obstruct pressurized steam infiltration. However, it is crucial to validate the suitability of any sterilization method before application (World Health Organization, 2011; Dion and Parker, 2013). Limitations of autoclaving include: not suitable for xenografts, as extreme heat and moisture may degrade biological tissues; primarily used for sterilizing scaffolds made from biopolymers, such as PCL and polyethylene terephthalate (PET) (Fracalossi Rediguieri et al., 2016).

One more physical sterilization method is dry heat sterilization. This method is more suitable for dehydrated solutions and dry powders but is not recommended for heat-sensitive materials. Standard dry heat sterilization conditions include: 170°C for 60 min, 160°C for 120 min, 150°C for 150 min (World Health Organization, 2011; Rutala and Weber, 2008). Like autoclaving, dry heat sterilization is not commonly used for scaffolds and is mainly applied to synthetic materials.

Radiation sterilization is another widely used physical sterilization approach (Tao et al., 2021). Two major techniques include gamma radiation and electron beam (E-beam) sterilization. Gamma radiation is the most widely used radiation-based sterilization technique. Its high penetration capacity, allowing sterilization through packaging and medical devices. Penetration depth is inversely proportional to material thickness (Booth, 2017). E-beam is ionizing radiation method with lower penetration depth than gamma rays, moreover unlike gamma sterilization, no post-treatment quarantine is required. This method is more suitable for materials that are sensitive to oxidative effects, as it may cause less damage than gamma radiation (Fracalossi Rediguieri et al., 2016).

Mechanism of radiation sterilization has two sides–direct and indirect effect. Direct effect based on disrupting nucleic acids, proteins, and enzymes of microorganisms, leading to loss of viability (Tao et al., 2021). Indirect effect induces radiolysis of water, generating free radicals that damage nucleic acids, proteins, and enzymes, ultimately impairing microbial metabolism (Tao et al., 2021; Booth, 2017). Advantages of radiation sterilization include performing at room temperature, preserving material integrity and high penetration ensures thorough sterilization (Fracalossi Rediguieri et al., 2016). Disadvantages of radiation sterilization are risk of protein denaturation and biopolymer degradation and possible changes in material color and mechanical strength (Dearth et al., 2016). Commonly radiosterilized dECM-derived tissues: heart valves (Helder et al., 2016), blood vessels (Goecke et al., 2018), liver (Mirmalek-Sani et al., 2013), stomach (Feng et al., 2020), bones (You et al., 2018) and tendons (Sun et al., 2020).

### Chemical sterilization methods

Ethylene oxide (EO) is a toxic chemical compound commonly used in the sterilization of scaffolds. EO, at room temperature, is a colorless gas with a pungent odor (Tao et al., 2021). However, it poses significant safety concerns: explosive in mixture with air in certain ratios and highly combustible and toxic to human (Fracalossi Rediguieri et al., 2016). EO functions as a good alkylating agent, interfering with microbial viability by denaturation of DNA and proteins as a result of alkylation and, besides, interfering with sulfhydryl, amino, and carboxyl groups on proteins and nucleic acids, leading to cell death (Fracalossi Rediguieri et al., 2016; Tao et al., 2021; Mendes et al., 2007). Despite its employment as a fine bactericidal, sporicidal, and virucidal agent, EO has some extraordinary limitations. EO toxicity requires thorough degassing upon sterilization, taking weeks (Fracalossi Rediguieri et al., 2016; Tao et al., 2021). EO is known as human carcinogen, and therefore there is a need for strict safety measures and specialized equipment for handling (Fracalossi Rediguieri et al., 2016). EO sterilization has been reported for dECM-derived tissues such as: blood vessels (Zhao et al., 2010), stomach (Dempsey et al., 2019), kidneys (Zhou et al., 2020), bones (Qing et al., 2019), tendons (Pan et al., 2015). Despite of some limitations, FDA (U.S. Food and Drug Administration) reports that EO sterilization is the most commonly used method to sterilize medical devices in the United States, and it is widely used by medical device manufacturers and contract sterilizers worldwide.

Peroxides constitute another group of commonly used chemical sterilant for biological materials. As strong oxidizers, peroxides are effective in incapacitating microbial structure. PAA and hydrogen peroxide (H_2_O_2_) are the most widely used peroxide-based sterilants.

PAA is very effective in killing bacterial spores under a short incubation period (∼30 min) (Rutala and Weber, 2008). Concentrations for use are normally 0.05%–1%. Materials are generally soaked in PAA solutions during the process of sterilization (Hussein et al., 2013). PAA oxidizes functional groups by electron transfer, leading to microbial cell death (Fracalossi Rediguieri et al., 2016) and attacks on cellular membranes, causing irreversible structural damage (Finnegan et al., 2010). The most important advantage of PAA sterilization is relatively safe, because its side products are acetic acid, water, oxygen, and carbon dioxide (Tao et al., 2021). At the same time, highly oxidative and acidic, which have the ability to alter the physical and chemical characteristics of certain materials (Fracalossi Rediguieri et al., 2016). PAA has been used to sterilize dECM-derived tissues, including: heart valves (Luo et al., 2014), liver (Sun et al., 2018), lungs (Zvarova et al., 2016), tendons (Jones et al., 2017).

H_2_O_2_ is a potent oxidizing agent applied primarily as a disinfectant. Under some conditions, it is sporocidal and therefore can be applied for sterilization (Hayrapetyan et al., 2020). Modified H_2_O_2_ sterilization exposure to radiation forms hydroxyl (•OH) and hydroperoxyl (HO_2_•) radicals, which enhance antimicrobial activity (Fracalossi Rediguieri et al., 2016). Low-temperature plasma sterilization (HPLP) is the low-temperature sterilization method most widely utilized in hospitals, operates at 45°C–55°C but never above 60°C (Tao et al., 2021). All these methods are rapid methods of sterilization and are completed in minutes. The main limitations of H_2_O_2_ and HPLP are gas embolism risk due to H_2_O_2_ reaction with catalase *in vivo*, producing oxygen, which may lead to embolism (Akuji and Chambers, 2017); strong oxidative effects that may disrupt protein tertiary structures, altering scaffold properties (Shah et al., 2018) and vacuum dependency as HPLP must be performed under vacuum conditions due to its low penetration capacity.

Among the various methods of sterilization, physical methods such as gamma radiation and electron beam sterilization are the most efficient and suitable for dECM-based scaffolds. They offer good penetration, can be done at room temperature, and result in minimal degradation of the materials and are therefore preferred to chemical sterilants.

### Biosafety of decellularized extracellular matrixes and scaffolds

Immunocompatibility is critical to the successful use of scaffolds in regenerative medicine because it determines the ability of implanted materials to be incorporated into tissues without triggering an adverse immune response. This is particularly critical in the context of xenogeneic scaffolds, where foreign antigens can result in rejection and inflammation. To prevent immune activation—most importantly, macrophage activation—and to promote greater tissue integration, there is a need to reduce the antigenicity of the material while preserving ECM structure (Abdul Samat et al., 2023; Fu et al., 2022; Hussein et al., 2016; Mansour et al., 2019).

Scaffold modification involves changes in their physic and chemical or biological properties to: a) increase biocompatibility by applying bioactive coatings or chemical surface modification to reduce immune response and improve cell adhesion; b) regulate mechanical properties–optimize stiffness, elasticity and porosity taking into account the requirements of recipient tissues; c) control microstructural organization: design architecture with appropriate size and connectivity of pores to ensure efficient transport of nutrients and metabolic products, as well as to direct cellular organization and growth (Bharathi et al., 2022).

Evaluation of scaffold biocompatibility includes *in vitro* and *in vivo* approaches. *In vitro* approaches involve laboratory assays for determining scaffold-cell and biological molecule interactions. They include cytotoxicity assays (e.g., MTT or MTS), which assess cell viability and cell proliferation support by scaffolds and determine toxic bioproducts. Cell adhesion and morphology analysis, using SEM and confocal laser scanning microscopy (CLSM), assess how cells spread and adhere on scaffold surfaces. Genotoxicity tests also assess whether scaffold materials induce DNA damage (Hussein et al., 2016; Kim et al., 2018; Viveros-Moreno et al., 2023).

*In vivo* methods involve implantation of scaffolds in living systems to assess general biocompatibility, including immune responses and host tissue integration. Histopathological analysis examines tissue at the implant site for inflammatory response, fibrotic response, and scaffold integration. Quantitative geometric analysis measure thickness of encapsulation and scaffold shape changes, and biodegradability and mechanical tests analyze long-term scaffold performance (Gong et al., 2018; Valencia-et al., 2022).

Studies confirm the huge potential of scaffolds in regenerative medicine. Shape-memory polymeric scaffolds enable bone tissue ingrowth with minimal inflammatory response, indicating excellent biocompatibility *in vivo* (Gasson et al., 2024). Gold nanoparticle-functionalized scaffolds have been found to reduce apoptosis marker expression and neutrophil recruitment, enhancing tissue compatibility and angiogenesis (Viveros-Moreno et al., 2023).

A number of scaffold modifications, such as adding extracellular matrix components and reducing immune stimulation, can potentially develop safer and more efficient tissue engineering materials.

### Scaffolds modifications

Natural scaffolds derived from animal or human tissues and organs more accurately replicate the native tissue microenvironment, thereby promoting appropriate cellular interactions. These scaffolds exhibit biocompatibility and biodegradability, making them suitable for biomedical applications (Neishabouri et al., 2022). One of the most promising types of natural scaffolds is the dECM, which serves as an optimal structural framework for tissue engineering due to the critical role of the ECM in tissue development (Gilbert et al., 2006).

ECM is water-containing composed of proteins—predominantly collagens—and polysaccharides (Kawecki et al., 2018). The organization and structure of ECM molecules, as well as microenvironmental factors (mechanical environment, pH, and CO_2_ concentration), vary depending on tissue type, function, and the specific cells responsible for ECM secretion (Frantz et al., 2010). Fibroblasts, adipocytes, and chondrocytes are among the key cell types involved in ECM formation, contributing to the production of structural proteins (e.g., fibronectins) and growth factors (Neishabouri et al., 2022).

Prior to clinical application, through implantation or transplantation, decellularized tissue undergoes a series of *in vitro* processing steps, including cell seeding, a process known as recellularization (García-Gareta et al., 2020). This pre-treatment enhances the potential for successful graft integration and function *in vivo*.

### Vascularization

Oxygen transport to tissues is a critical factor in regeneration and transplantation. The application of dECM is limited by its thickness, because inadequate oxygen diffusion into the scaffold core may undermine cell survival. Optimizing the maintenance of ECM components and their vascular structure maximizes post-implant perfusion, thereby enhancing angiogenesis. For instance, basal membrane components such as laminin and fibronectin play a crucial role in tissue revascularization (García-Gareta et al., 2020; Partington et al., 2013).

Vascularization can be induced through the incorporation of angiogenic factors into the matrix or by prevascularizing the dECM before implantation (Amirsadeghi et al., 2020). Since bone grafts are the second most commonly transplanted tissues after blood transfusions (Wang and Yeung, 2017), significant research has been dedicated to overcoming the challenges of large-scale bone defect reconstruction. Inflammatory responses triggered by dECM-based bone grafts may induce spontaneous blood vessel formation (Yi et al., 2016). However, the relatively slow revascularization of transplanted tissue remains a key limitation factor in the regeneration of critical-sized defect healing (Mokhtari-Jafari et al., 2020). Prevascularization is facilitated by the induction of recruitment of host cells and collagen deposition by increased levels of fibrinogen and expression of connective tissue growth factor, initiating direct new vessel formation (Cheng et al., 2018).

Growth factors play a central role in vascularization since they affect endothelial cell migration, proliferation, and aggregation directly or indirectly (Sun et al., 2021). Currently, the primary angiogenic growth factors used to promote dECM properties belong to three general approaches (Lv et al., 2024).

Direct targeting of endothelial cells employs biological agents that directly stimulate endothelial cells and thus trigger angiogenesis and tissue repair. The key players involved here are vascular endothelial growth factor (VEGF) and angiopoietin. VEGF is crucial during the phase of early vascularization, triggering the formation of a native vasculature. Angiopoietin, on the other hand, regulates vascular remodeling and maturation to achieve structural integrity in new vessels. Use of broad-spectrum growth factors and chemokines employs factors that are active on several endothelial cell types, which enormously enhances vascularization. Fibroblast growth factor (FGF) is a very good example, which stimulates endothelial cell proliferation and chemotaxis and thus speeds up the angiogenic process.

Indirect angiogenesis induction approach involves factors that indirectly promote angiogenesis by leading to the release of pro-angiogenic signals. TGF-β is among the key players in this group, as it maintains vascular wall integrity, while platelet-derived factors induce smooth muscle cell proliferation, helping in vessel maturation and stabilization (Lv et al., 2024; Li et al., 2020; Ka et al., 2021).

These strategies offer promising directions for improving dECM-based graft integration, ultimately leading to the advancement of tissue engineering and regenerative medicine.

### Immunogenicity and strategies for reducing host immune response

All non-autologous material containing foreign elements has the potential to trigger an immune response in the host organism. The immune response can lead to severe complications, including transplant rejection. Activation of the immune system against synthetic, biologic, and decellularized xenogeneic and allogeneic grafts therefore represents one of the major challenges for transplantation of engineered organs and tissues (Wiles et al., 2016).

Multiple techniques have been developed to suppress the immune response of the recipient, including scaffold surface modification via immunomodulatory coatings, immune masking, protein modification that targets damage-associated molecular patterns (DAMPs), and recellularization using autologous cells to block immune activation (Wiles et al., 2016). Of these, recellularization of the scaffold has emerged as an extremely promising and highly studied technique. This approach allows for the use not only of the patient’s own cells but also gene-modified hypoimmunogenic stem cells to prevent immune rejection (Deuse et al., 2019).

Another significant dECM use limitation is that most of the current information is derived from animal models. This makes it difficult to accurately extrapolate human immune response from the animal’s immunological response. In an effort to meet this void, non-human primates and humanized mouse models with more analogous immune systems to the human immune system are being actively explored to more clearly understanding the immunogenic components of dECM (Bilodeau et al., 2020).

### Surface modification of decellularized scaffolds for enhanced biocompatibility

For improving cytocompatibility, mechanical strength, and biological activity and minimizing inflammatory reactions, decellularized scaffolds can be surface-modified using various techniques and biomaterials (Liu et al., 2021).

The chemical characteristics of the scaffold surface play a crucial role in enhancing biological properties by ensuring a hydrophilic interface, which promotes the adhesion of diverse materials and improving cell attachment. Chemical functionalization enables the incorporation of functional groups on the scaffold surface, enabling immobilization of polymers, nanoparticles, biomolecules, bioactive compounds, and inorganic compounds. These molecules bind to the scaffold surface through covalent bonds, which are extremely stable and resistant to degradation, hence improving the properties and overall performance of the dECM. Methods for the enhancement of the chemical functionality of dECM can be classified into two main types: native chemical modification and covalent chemical modification.

First technique involves the grafting of functional groups such as hydroxyl (-OH), amine (-NH_2_), and carboxyl (-COOH) onto the surface of polyester-based scaffolds. The resulting grafting reactions create an active interface, allowing for interactions with other materials. A second method involves the utilization of linker molecules that undergo stable covalent bonding, allowing surface functionalization to be controlled (Teimouri et al., 2023).

As mentioned earlier, one of the most critical scaffold modifications is recellularization, which significantly enhances scaffold properties. The most crucial purpose of surface modification of tissue scaffolds is enhancing cell adhesion and proliferation, which are depend on scaffold hydrophilicity. Hydrophilic surfaces offer the most effective cell adhesion. Physical adsorption and chemical modification are two principal methods of scaffold surface modification. Physical method involves soaking or immersing the scaffold in a medium containing biological molecules or bioactive molecules. These agents adhere to the surface of the scaffold through electrostatic forces, hydrogen bonding, and Van der Waals forces. Physical adsorption is less used due to its instability (Yoshida et al., 2006). Chemical method includes a variety of techniques, such as: plasma treatment using different gases to alter surface characteristics (Hesari et al., 2021); chemical cross-linking by means of agents such as genipin (Tonda-Tur et al., 2011) and glutaraldehyde (Nieuwoudt et al., 2021); functionalization using chemical linkers such as dopamine (JináLee et al., 2018), silane coupling agents (Ghorbani et al., 2020), and 1,6-hexamethylenediamine (Mohammadi et al., 2018).

These modification strategies significantly enhance scaffold bioactivity, ensuring improved integration and performance in tissue engineering applications.

### Recellularization of decellularized scaffolds

*In situ* tissue regeneration leverages the regenerative potential of the recipient’s body, making pre-seeding of cells onto dECM before transplantation unnecessary. The regeneration process can, nevertheless, be enhanced with the addition of bioactive molecules (Yang et al., 2020). Despite this advantage, dECM may possess thrombogenic and immunogenic determinants on their surface, which may lead to complications such as thrombogenesis and acute immune responses. These adverse reactions can be prevented or shielded if cells are adhered on the surface of the scaffold.

Recellularization is a dynamic process in which decellularized organ scaffolds are seeded with patient-specific cells either *ex vivo* or *in vivo* within a high nutrient media. Successful recellularization of whole organs requires an appropriately designed environment that mimics the physiology of the target organ. This procedure is usually divided into two phases: static cell culture–initial phase where cells attach and proliferate on the scaffold surface, dynamic recellularization–a later phase where perfusion of scaffold with a suspension of cells within a nutrient solution is performed to favor cell spread and integration (Stoian et al., 2024).

The dynamic recellularization stage consists of perfusion of the scaffold with a cell suspension to increase cell penetration and scaffold colonization (Hillebrandt et al., 2019). Bioreactors are some of the most effective systems for maximizing recellularization quality. Most bioreactors utilize perfusion-based systems that yield controlled fluid flow. These systems improve recellularization by subjecting the cells to gravitational forces, rotation cultures, optimal oxygen levels, or mechanical stress to simulate the endogenous microenvironment of the tissue, e.g., compression (Selden et al., 2018). Rotating flasks, rotating cylindrical vessels, perfusion bioreactors and microfluidic devices are some of the most commonly used bioreactor types (Ahmed et al., 2019). Bioreactors are designed to meet the technical and biological requirements of specific organs and enable scaffold growth under optimal conditions (Chen and Hu, 2006). Compared to static cell culture systems, bioreactors provide precise control of environmental parameters. The incorporation of sensors integrated within the system enables the measurement of changes such as pH levels or concentration levels of specific bioactive factors (Simmons et al., 2017).

During and after recellularization, it is essential to monitor scaffolds to optimize and control culture conditions. Therefore, a bioreactor should be capable of tracking key biological parameters—such as temperature (optimal for mammalian cell lines is 37°C), pH, oxygen levels, CO_2_ levels, glucose consumption, and lactate production—ideally in real time and using non-invasive methods (Mahfouzi et al., 2021). Mammalian cell lines function best at a physiological range of pH from 7.0 to 7.4, maintained by a bicarbonate buffer system. pH must be continuously monitored, and CO_2_ is added to decrease it when necessary. Conversely, to raise the pH, oxygen is introduced into the sparger to remove dissolved CO_2_. Oxygen can be supplied by sparging air, a mixture of air and O_2_, or pure O_2_ (pO_2_), typically via a sparger positioned below the impeller. However, when lactate accumulates in the medium, simply adding air is insufficient. In such cases, a basic solution—such as 0.five to one M NaOH or Na_2_CO_3_—may be added to restore the desired pH (Collignon, 2020).

Factors such as fluid flow rate, nutrient distribution, and flow-induced shear stress within bioreactors can significantly influence experimental outcomes. Developing appropriate and reproducible protocols requires careful adjustment of bioreactor parameters to match the specific cell type and scaffold architecture. This process is often complex, time-consuming, and resource-intensive, frequently involving trial and error. Integrating mathematical modeling with advanced bioprocessing infrastructure may help streamline the development of functional grafts by predicting and optimizing effective bioreactor configurations, ultimately improving tissue engineering outcomes (Nokhbatolfoghahaei et al., 2020).

In addition to bioreactor-based techniques, there are also alternative recellularization methods. The overall strategy of fragment-based recellularization in the following steps: small pieces of scaffolds (∼1 cm^2^) are placed into 24-well plates, seeded with a cell suspension, and maintained under slow rotation. Mild mixing over several hours allows cell suspension infiltration into scaffold structure (Gardin et al., 2015; Zhou et al., 2022). Direct infusion of concentrated cell suspension in small volume directly into scaffold with incubation in routine culture condition until cells reached confluency is another simple method of scaffold seeding (Shchotkina et al., 2021; Sokol et al., 2020).

Recellularization presents several limitations. First, it is a time-consuming process, particularly when using induced pluripotent stem cells (iPSCs) derived from a patient’s somatic cells. Another important factor is the need for sufficient cell numbers of the appropriate type. Since seeding density of cells depends on the type of cell and reconstructed tissue, this factor must be strictly controlled to limit the risk of teratoma development or immune activation (Kim et al., 2020).

Stem cells are the most commonly used cell types for recellularization due to their high proliferative capacity. They are generally classified into the following categories: adult stem cells, embryonic stem cells (ESCs), fetal stem cells and iPSCs (Bacakova et al., 2018). Of all these, the most frequently applied stem cells for recellularization are mesenchymal stem cells (MSCs) and hematopoietic stem cells (HSCs). ESCs hold the highest differentiation potential, but their use is restricted due to ethical concerns and challenges in accessing them. iPSCs, which are derived through genetic reprogramming of adult somatic cells (Moser et al., 2014), possess histocompatibility advantages and avoid ethical concerns but whose use is limited due to the risk of tumorigenicity. MSCs are multipotential and easily accessed from tissues such as bone marrow and fat tissue. They can differentiate into various cell lineages and exhibit potential in the recellularization of other tissues.

### Future directions of scaffold production

Manufacturing scaffolds of non-animal origin is a is one of the most popular research areas in the field of bioengineering. Such scaffolds have the potential to avoid limitations associated with decellularization.

Synthetic polymers play an important role in scaffolding due to the ability to precisely control their properties and scale up production. Key synthetic polymers used in scaffolding include: a) poly(lactide-co-glycolide) (PLGA) - a biodegradable polymer with controlled degradation, is a promising material for the regeneration of bone, skin and nervous tissues; b) poly(ε-caprolactone) (PCL) – is characterized by high biocompatibility and slow degradation, which makes it effective for long-term implants, for example, in orthopedics; c) polyurethanes and polyesters–are used in scaffold production due to their elasticity, mechanical strength and tunable properties (Brink, 2021; Dhand et al., 2016; Sola et al., 2019).

Non-animal scaffold fabrication techniques include solvent casting and particle leaching, freeze-drying, thermally induced phase separation, gas foaming, powder processing, sol-gel synthesis, and electrospinning.

Particle leaching and solvent casting require dissolving a polymer in a solvent that has well-dispersed salt particles of a specific size. Solvent evaporation results in a polymer matrix with incorporated salt particles that are then leached out in aqueous solution to produce a porous network. This method provides scaffolds of high porosity and adjustable pore sizes (Thavornyutikarn et al., 2014).

An example of solvent casting and particle leaching is the use of scaffolds made from polymethyl methacrylate (PMMA) and polyurethane (PU) to mimic the *in vivo* bone marrow (BM) microenvironment for the study of stromal cell behavior. In an experiment (Sola et al., 2019), human stromal cells (HS-5) were seeded on collagen-coated PMMA and PU scaffolds for successful cell adhesion and growth with maintaining the porous structure of the support materials. The connectivity of the porous network enabled cell migration to the interior of the scaffold. Immunofluorescent staining confirmed that cells not only adhered onto the scaffold surface but even migrated into the connected pores, with a morphology of fibroblast-like cells and a highly ordered cytoskeleton. Collagen coating did not alter the pore architecture of the material, and cells were capable of colonizing both PU and PMMA supports.

Lyophilization or freeze-drying is another scaffold fabrication method. A polymer is dissolved in solvent and frozen under temperature below the melting point, which causes the solvent to solidify. The solvent is sublimated to produce a dry scaffold with numerous connected pores. The advantage of this method lies in avoiding the use of high temperatures, which maintains the activity of biological factors. It is possible to control freezing conditions to regulate pore size. It has been used to prepare a bioactive glass-collagen-phosphatidylserine scaffold with 300-µm pores that are not isolated. Phosphatidylserine reacts with phosphate and calcium to form hydroxyapatite crystal nuclei (Chen et al., 2015). It does have the drawback of long processing time, high energy consumption, use of cytotoxic solvents, and limited pore sizes (15–35 µm). The addition of conditions such as freezing point changes and adding annealing procedures has increased pore diameters to 85–325 µm (Bajaj et al., 2014).

Gas foaming was developed as a replacement for cytotoxic solvents. It is a method using inert gases (e.g., carbon dioxide, nitrogen) to saturate water or fluoroform-activated biodegradable polymers and form gas bubbles. The process tends to generate sponge-like matrices with up to 85% porosities and pore sizes of 30–700 µm (Thavornyutikarn et al., 2014). A polyurethane mandibular bone defect healing scaffold contained a dense surface layer and a porous core to enable bone regeneration. Mechanical testing confirmed its ability to withstand implantation loads (Giannitelli et al., 2015). While gas foaming is beneficial, it suffers from some restrictions like excessive heat generation during pressing, discrete pores, and lack of a porous surface layer.

Sol-gel processing originates from inorganic polymerization of metal alkoxides. Sol stage is developed by the addition of surfactants that promote condensation and gelation. Sol-gel processing can yield ceramic or glassy materials in various forms, including powders, coatings, fibers, membranes, and aerogels (Thavornyutikarn et al., 2014). A study (Ma et al., 2022) compared the cytocompatibility of chitosan-based composite scaffolds that were fabricated via sol-gel synthesis. MC3T3-E1 cells cultured on these scaffolds showed good viability and adhesion success. CCK-8 assay showed active cell proliferation, and staining indicated most cells were viable, indicating good support for growth. These composite materials had optimum porosity and mechanical properties, and they supported cell growth.

Electrospinning utilizes electric charges to draw nanoscale polymer fibers through a syringe pump, creating a nanofibrous structure capable of adsorbing proteins and binding to cell membrane receptors. The typical setup includes a spinneret with a metal needle, a syringe pump, a high-voltage power supply, and a grounded collector. The electric field overcomes the surface tension of the polymer droplet, forming a charged liquid jet that stretches and undergoes electrostatic whipping before being deposited onto the collector. The evaporation of the solvent solidifies the jet, forming a nonwoven fibrous membrane. The technique provides flexibility in the production of scaffolds with different morphology and porosities, ranging from nanometers to micrometers (Bajaj et al., 2014).

## Conclusion

This review determines the rationale behind the selection of various decellularization protocols for various tissues. The chosen decellularization strategy must be sufficiently effective to remove all immunogenic components from the dense tissue while preserving essential ECM components such as glycosaminoglycans, collagens, and growth factors. Physical methods, in general, cause the least damage to cells. Effective removal of cell material, however, typically requires the use of chemical methods. Chemical treatment alone, on the other hand, is insufficient for effective decellularization due to the limited diffusion of reagents into cells. Physical methods provide the entrance for chemical agents, enhancing the overall efficiency of the process. Therefore, most decellularization protocols employ a synergistic combination of physical, chemical, and enzymatic methods.

For effective and secure clinical use of scaffolds, sterilization plays a significant role. As discussed in the literature, irradiation—gamma rays or electron beam exposure—are the most widely used physical methods of sterilization. These have been found to be very effective and are suitable for bio-scaffolds as they do not cause significant structural damage or loss of mechanical properties. Chemical sterilization processes also possess potent antimicrobial activity. They can be too harsh, however, and if trace quantities of sterilizing agents are not properly eliminated, can cause scaffold cytotoxicity. Additionally, some chemical treatments necessitate special equipment and have carcinogenic or toxicity risks associated with them for the human operator.

Scaffolds modifications methods can help to enhance biocompatibility and implantation of final product. The most effective way to control recipient immune response is recellularization of dECM. Successful recellularization of whole organs requires an appropriately designed environment that mimics the physiology of the target organ. Moreover, the regeneration process, as well as preventing thrombogenesis and acute immune responses, can be prevented or shielded if cells are adhered on the surface of the scaffold.

## Abbreviations

*BM*, bone marrow; *CHAPS*, 3-([3-cholamidopropyl]dimethylammonio)-1-propane sulfonate hydrate; *CLSM*, confocal laser scanning microscopy; *DAMP*, damage-associated molecular patterns; *dECM*, decellularized extracellular matrix; *E-beam*, electron beam; *ECM*, extracellular matrix; *EDTA*, ethylenediaminetetraacetic acid; *EGTA*, ethylene glycol tetraacetic acid; *EO*, ethylene oxide; *ESC*, embryonic stem cell; *FGF*, fibroblast growth factor; *GAGs*, sulfated glycosaminoglycans; *HHP*, high hydrostatic pressure; *HPLP*, low-temperature plasma sterilization; *HSC*, hematopoietic stem cell; *iPSC*, induced pluripotent stem cell; *MSC*, mesenchymal stem cell; *NTIRE*, non-thermal irreversible electroporation; *PAA*, peracetic acid; *PCL*, polycaprolactone; *PET*, polyethylene terephthalate; *PLGA*, poly(lactide-co-glycolide); *PMMA*, polymethyl methacrylate; *PU*, polyurethane; *SDS*, sodium dodecyl sulfate; *SDC*, sodium deoxycholate; *SEM*, scanning electron microscopy; *TBP,* tributyl phosphate; *TGF-β*, transforming growth factor-β; *VEGF*, vascular endothelial growth factor.
